# Cross-platform analysis of atrial fibrillation scientific videos: using composite index and a basic assessment scale

**DOI:** 10.3389/fpubh.2025.1507776

**Published:** 2025-04-25

**Authors:** Chong Luo, Xiaoli Qin, Xiaoyu Xie, Jie Gao, Yuwei Wu, Weitao Liang, Zhong Wu

**Affiliations:** ^1^Department of Cardiovascular Surgery, West China Hospital, Sichuan University, Chengdu, Sichuan, China; ^2^Department of Anesthesia, West China Hospital, Sichuan University, Chengdu, Sichuan, China; ^3^West China School of Medicine, Sichuan University, Chengdu, Sichuan, China; ^4^West China School of Public Health, Sichuan University, Chengdu, Sichuan, China

**Keywords:** patient education, atrial fibrillation, social media platform, author type, video content quality

## Abstract

**Background:**

Currently, video platforms were filled with many low-quality, uncensored scientific videos, and patients who utilize the Internet to gain knowledge about specific diseases are vulnerable to being misled and possibly delaying treatment as a result. Therefore, a large sample survey on the content quality and popularity of online scientific videos was of great significance for future targeted reforms.

**Objective:**

This study utilized normalization data analysis methods and a basic assessment scale, providing a new aspect for future research across multiple platforms with large sample sizes and for the development of video content quality assessment scales.

**Methods:**

This cross-sectional study analyzed a sample of 331 videos retrieved from YouTube, BiliBili, TikTok, and Douyin on June 13, 2024. In the analysis of atrial fibrillation scientific videos across four social media platforms, comprehensive metrics and a basic scoring scale revealed associations between platforms, creators, and the popularity and content quality of the videos. Data analysis employed principal component analysis, normalization data processing, non-parametric tests, paired t-tests, and negative binomial regression.

**Results:**

Analysis of the user engagement data using a composite index revealed a significant difference in the popularity of videos from publishers with a medical background (z = −4.285, *p* < 0.001), no aforementioned findings were found among video platforms, however, except for the Bilibili platform. As for content quality, while the difference in the total number of videos between the two groups was almost 2-fold (229:102), the difference in qualified videos was only 1.47-fold (47:32), a ratio that was even more unbalanced among the top 30% of videos with the most popularity. Notably, the overall content quality of videos from publishers without a medical background was also significantly higher (z = −2.299, *p* = 0.02).

**Conclusion:**

This analysis of atrial fibrillation information on multiple social media platforms found that people prefer videos from publishers with a medical background. However, it appeared that these publishers did not sufficiently create high-quality, suitable videos for the public, and the platforms seemed to lack a rigorous censorship system and policy support for high-quality content. Moreover, the normalized data processing method and the basic assessment scale that we attempted to use in this study provided new ideas for future large-sample surveys and content quality review.

## 1 Introduction

Atrial fibrillation (AF) is the most common clinical arrhythmia, affecting approximately 59 million individuals worldwide (1). Patients with AF face a 2-fold increased risk of myocardial infarction (2) and a 5-fold increased risk of heart failure (3). Additionally, they have a 5-fold higher risk of ischemic stroke, which rises to 20-fold in the presence of mitral stenosis (4, 5). According to Lin et al., 28.6% of AF patients are asymptomatic and often hospitalized for other primary conditions (6). Therefore, educating patients about the health risks associated with AF may improve adherence to treatment more effectively than focusing solely on medication dosages and routine follow-ups.

Traditional medical education methods, such as posters and manuals, are often limited in their reach due to accessibility and geographic constraints (7). This restricts the dissemination of disease-related information to both chronic disease patients and the public (6, 8, 9). Today, with the widespread use of smartphones and personal computers, individuals can access health information anytime and anywhere. This shift has made online video platforms a powerful tool for distributing medical education and raising awareness (10–12). Many healthcare institutions on platforms like YouTube have obtained certification and are actively publishing videos (13). Lee et al. found that 40.8% of U.S. adults use YouTube to search for health-related information, leading to a 30% increase in physical activity (14). However, some meta-analysis of educational videos on online platforms revealed that there is still considerable room for improvement, particularly in terms of content accuracy and regulatory oversight on platforms like YouTube (15–17). Platforms demonstrate inadequate content moderation for user-uploaded videos, enabling virtually any user to disseminate disease-related articles or videos without rigorous vetting. Coupled with social media’s intrinsic real-time nature that facilitates viral propagation speeds, misinformation, unverified claims, and pseudoscientific content become freely accessible through platform search algorithms (15, 18, 19), The insufficient creator-audience interaction diminishes the signature dialogic nature of social media platforms (20), effectively rendering them functionally equivalent to traditional didactic health broadcasts.

Current research on the quality of educational videos predominantly consists of single-platform, single-disease studies with small sample sizes and video quality evaluations rely on horizontal comparisons of isolated unidimensional metrics (e.g., view counts, likes), without in-depth exploration of inter-indicator correlations. There is a notable lack of cross-platform quality comparative analyses and disease-specific quality assessment tools (depending on generic scales like DISCERN or JAMA). In this study, AF educational videos were retrieved from four platforms: YouTube, BiliBili, TikTok, and Douyin. We experimentally applied a normalization method to unify interaction data from different platforms for statistical analysis and used a ‘basic and essential’ professional rating scale to assess the content quality of sampled videos. This study provided a new perspective and method for future large-scale data analysis and content quality evaluation across different platforms.

## 2 Methods

### 2.1 Study design

This study was a cross-sectional analysis. On June 13, 2024, we performed keyword searches for “Atrial Fibrillation,” “AF,” and “AF + Management” across YouTube, BiliBili, TikTok, and Douyin platforms. For each video identified, we recorded the URL, the number of views, likes, and the number of comments and replies on the same day to mitigate potential changes over time. The content quality of the videos was then evaluated over the subsequent month.

### 2.2 Measures

The keyword searches were independently conducted by two reviewers using a web browser with a cleared cache. The top 50 results for each search term were selected. Exclusion criteria included duplicate videos, audio-only content, videos with titles that did not match the content, and videos in languages other than English or Chinese. In cases where the two reviewers disagreed, a third reviewer was consulted to make the final decision. After applying these criteria, a total of 331 videos were included in the analysis.

### 2.3 Data collection

#### 2.3.1 Interaction metrics

**Figure 1:**
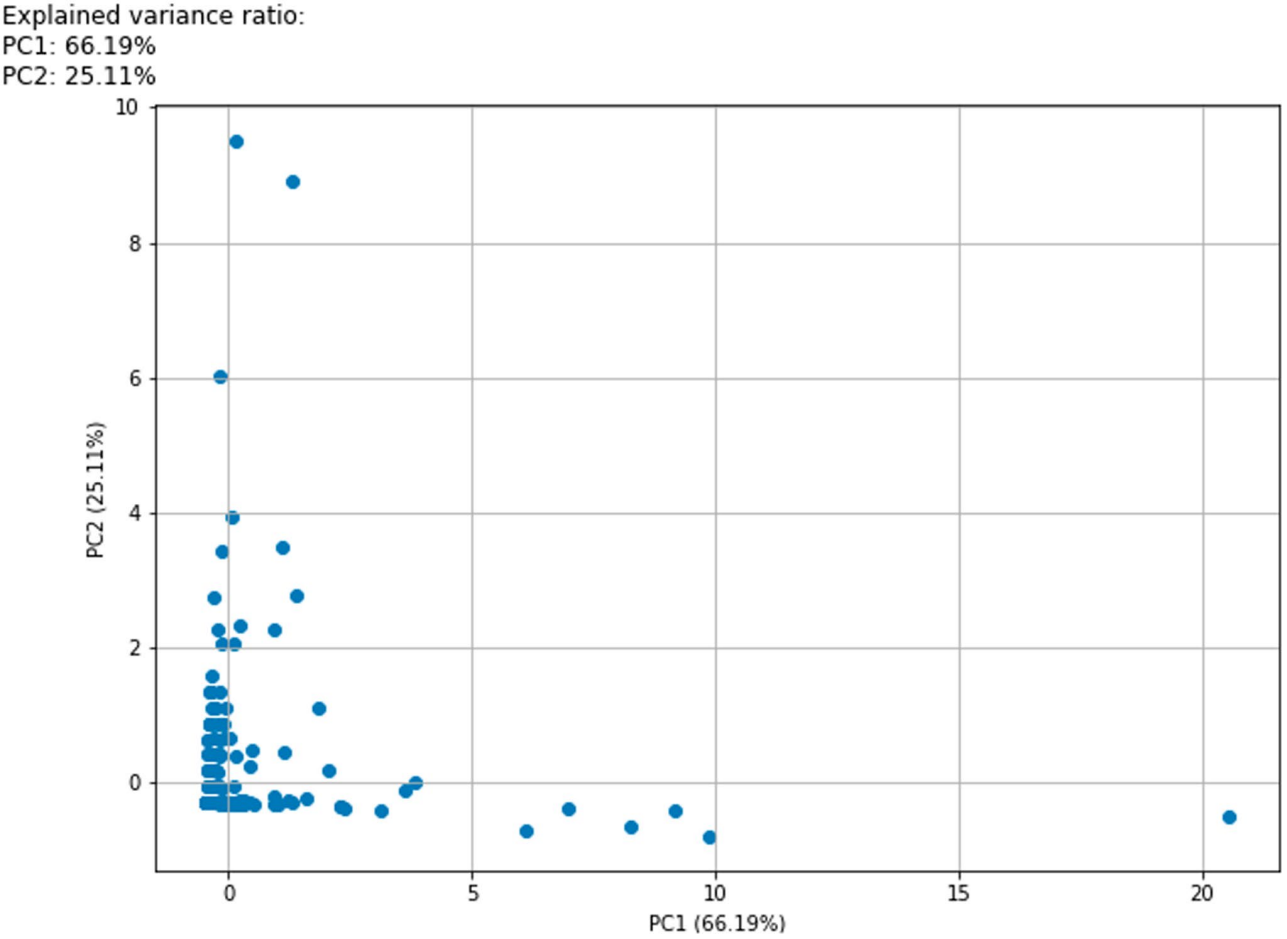
Interaction metrics PCA 2D scatter plot. The horizontal and vertical coordinate are the principal component (PC1,2) axis and principal component contributions. PC1 explains 66.19% of the variance in the dataset. Specific data for each principal component loading can be found in Table 1B. Blue dots represent each sample included in the analysis.

The interaction metrics collected for each video included: view count, number of likes, comments, and replies. To avoid potential bias, we matched 1:1 after data collection in rows stratified by a reply/comment ratio of 3:1. Metrics between different platforms were examined using Kruskal-Wallis tests (Table 1A). Principal Component Analysis (PCA) and weighted scoring were employed to preprocess interaction metrics data [Kaiser-Meyer-Olkin (KMO) = 0.741; and Bartlett’s test of sphericity < 0.001]. Based on the PC1 loading, the Heatscore, a composite index, was obtained using a normalization method (Figure 1; Table 1B).

**Table 1A:** Interaction metrics categorized by platforms.

Platform	Views	Likes	Comments	Replies
Bilibili (*n* = 100)	1557.0 (456.5,8927.5)	17.5 (7.0,172.5)	2.0 (0.0,15.8)	0.0 (0.0,0.0)
Tiktok (*n* = 79)	41200.0 (16600.0,124300.0)	1369.0 (344.0,3469.0)	44.0 (19.0,165.0)	1.0 (0.0,4.0)
YouTube (*n* = 49)	40825.0 (9285.5,173419.5)	485.0 (92.5,1910.5)	11.0 (1.0,81.5)	0.0 (0.0,0.0)
Douyin (*n* = 103)	27822.3 (8464.5,62007.8)	1628.0 (517.0,3623.0)	117.0 (39.0,266.0)	0.0 (0.0,0.0)
*p* value	0.000**	0.000**	0.000**	0.000**

*p < 0.05; **p < 0.01; Kruskal-Wallis test.

**Table 1B:** PCA loadings.

	PC1	PC2	PC3	PC4
Views	0.57	0.05	0.79	0.22
Likes	0.59	0.03	0.20	0.78
Comments	0.58	0.06	0.57	0.58
Replies	0.02	1.00	0.08	0.00

#### 2.3.2 Specialized metrics

The DISCERN and JAMA scales have been utilized to evaluate the accuracy of information sources. However, the aforementioned scales are capable of assessing the reliability of the information sources present in the video but offer a limited capacity for the evaluation of content richness on a professional area. This observation is consistent with the findings of previous studies that have expressed reservations regarding the application of the DISCERN and JAMA scales (21–23), Therefore, the classification criteria developed by clinicians were used in this study. A scoring system, the Atrial Fibrillation Specific Score (AFSS), was designed based on existing literature and expert guidelines (24, 25). However, using such complex and precise scales to guide the creation of educational videos may result in content that is too advanced for viewers to comprehend, limiting their ability to engage in shared decision-making (26). In the course of routine treatment, it has been observed that patients tend to demonstrate a limited interest in the pathogenesis, preferring instead to inquire about the treatment options and the distinctions between them. However, during outpatient consultations, patients frequently encounter challenges in comprehending and assimilating a substantial volume of disease-related information within a limited timeframe. This observation underscores the imperative for the development of a scoring scale that quantifies the quality of popularized video content, with the objective of enhancing public comprehension of medical information. On the one hand, it can effectively promote the platform’s content review, and on the other hand, we hope that video authors can produce videos based on this scale to provide a good way for the public to understand and absorb disease-related knowledge before and after the treatment. To address this, we developed the Essential Score (Escore) as a simplified version of the AFSS framework. We have attempted to remove from the AFSS framework about diagnosis and management of the disease that may require a medical background to understand, and to focus on knowledge that is of more interest to the general public, such as the course of the disease, its associated dangers, methods for self-diagnosis, and available treatment options. Five specialized cardiac surgeons from our hospital were consulted to evaluate the content validity of both the AFSS and Escore rating scales. After multiple rounds of adjustments and assessments, the final criteria are presented in Table 2. Each video was scored for content quality independently by three authors, or by the corresponding author if the variation between them was more than three. The reliability, validity, and content validity indexes confirmed that these scales are appropriate for evaluating the content quality of the videos analyzed in this study (Table 2).

**Table 2:** Specialized metrics scales.

Diagnose	Treatment	Management
1. Atrail Fibrillation Defination ***(Essential)***	1. Anticoagulant/Avoid stroke ***(Essential)***	1. Discussion of the indications/contradictions/possible complications of various treatments
2. Risk factors for AF	2. Surgical options such as AF ablation procedures(catheter/surgical)/left atrail appendage ***(Essential)***	2. Notifications of postoperative or drug follow-up care
3. Common clinical manifestation ***(Essential)***	3. Antiarrhythmic drug therapy ***(Essential)***	3. Mentions of AF is a disease that requires long- term treatment and needs patients’ own participation in the management of this disease ***(Essential)***
4. Findings of a physical examination		4. Attentions to the prevention and control of risk factors of AF
5. Transthoracic echocardiography as diagnostic technique		
6. The consequences of allowing AF to be left untreated ***(Essential)***		

Concordance between the three authors: Fleiss Kappa = 0.765. Escore reliability: Cronbach’s a = 0.715; Validity: Kaiser-Meyer-Olkin (KMO) = 0.746; and Bartlett’s test of sphericity <0.001; Content validity: S-CVI/UA = 0.857, S-CVI/Ave = 0.971.

In this study, the content quality of the sample videos was evaluated using the Escore with one point awarded for fulfilling one item in the Diagnose and Management section, but slightly different in the Treatment section. With a clear diagnosis of a chronic disease, there is mostly an emphasis on long-term treatment and regular follow-up (27–29), and it is important for patients to have a thorough understanding of the treatments for their condition (30, 31), so a missing item in the Treatment section of the Escore was worth zero points. In video quality grading, treatment videos scoring below 3 were classified as disqualification. Scores of 5–7 and 3–4 were classified as adequacy and eligibility.

### 2.4 Statistical analysis

All videos were categorized based on publishing platform and author type. Categorical variables were analyzed using frequency and relative frequency, whereas continuous variables were summarized using median values. Python was utilized to perform PCA and calculate the Heatscore index. Pandas and NumPy were employed for data processing and numerical computations, PCA and the calculation of the Heatscore. IBM SPSS Statistics version 26.0 was applied for statistical analysis and the computation of the metrics. According to the Kolmogorov–Smirnov test, the data in this study exhibited non-normal distribution, necessitating the use of non-parametric tests to evaluate differences between groups.

## 3 Results

Out of the initial pool of 600 videos, 269 were excluded due to duplication, lack of audio, or irrelevance. This left 331 videos for analysis, with 31% from non-medical backgrounds (NMB), such as self-media and public accounts, and 69% from medical backgrounds (MB), including doctors and hospital media.

**Figure 2:**
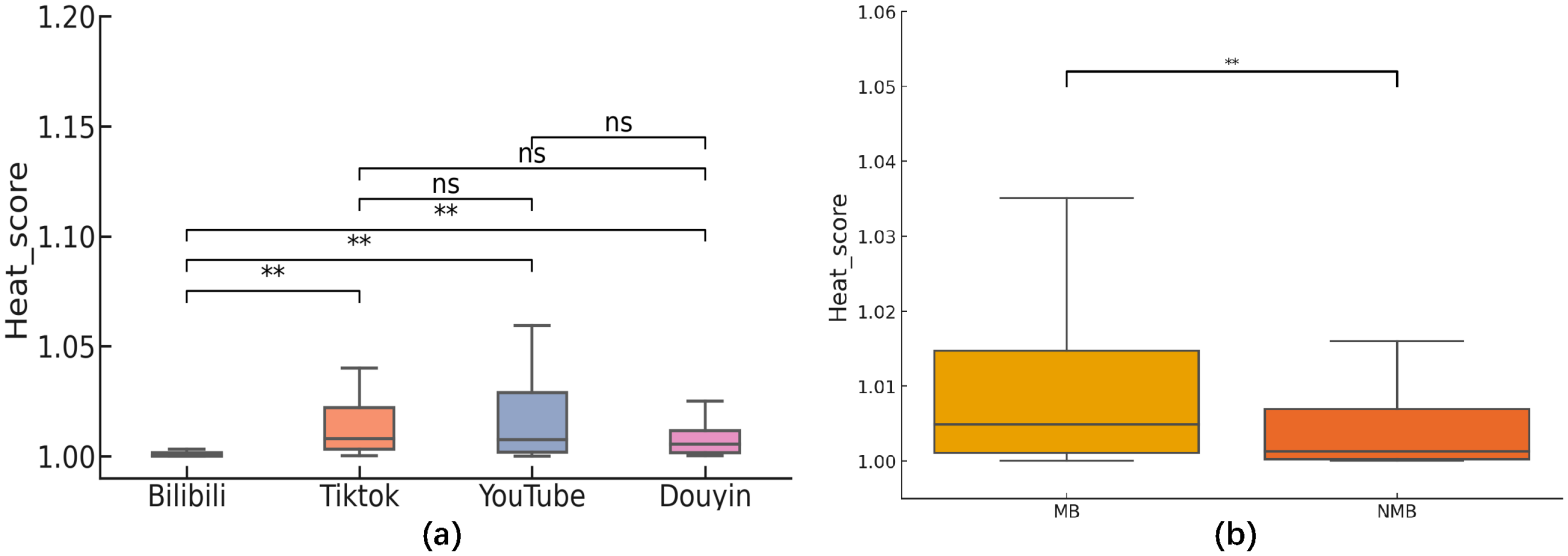
**(a)** Analysis of between-group differences in heat score categorized by platform. The results of the two-by-two comparison based on the Nemenyi test showed that there were statistically significant differences between the observed metrics of the Bilibili platform and the other three analyzed platforms (TikTok, YouTube, and Douyin; *p* < 0.001). Comparisons between the other platforms were as follows: the difference between TikTok and YouTube was not statistically significant (*p* = 0.983), nor was the difference with Douyin (*p* = 0.405); the comparison between YouTube and Douyin also showed no significant difference (*p* = 0.779). **(b)** Analysis of between-group differences in heat score categorized by author type. The MB group had higher heat score then the NMB group (z = −4.285, *p* < 0.001).

In the first phase, we analyzed interaction metrics (views, likes, comments, and replies) and specialized metrics (AFSS and Escore) based on video platform and author type. In the interaction metrics section, after processing through PCA and weighted scoring, we found that the Heatscore still showed significant differences among various video platforms. A subsequent Nemenyi post-hoc test revealed that the source of the difference was solely the video data from the Bilibili platform (Figure 2a). The MB group had higher Heatscore than the NMB group (z = −4.285, *p* < 0.001; Figure 2b).

**Figure 3:**
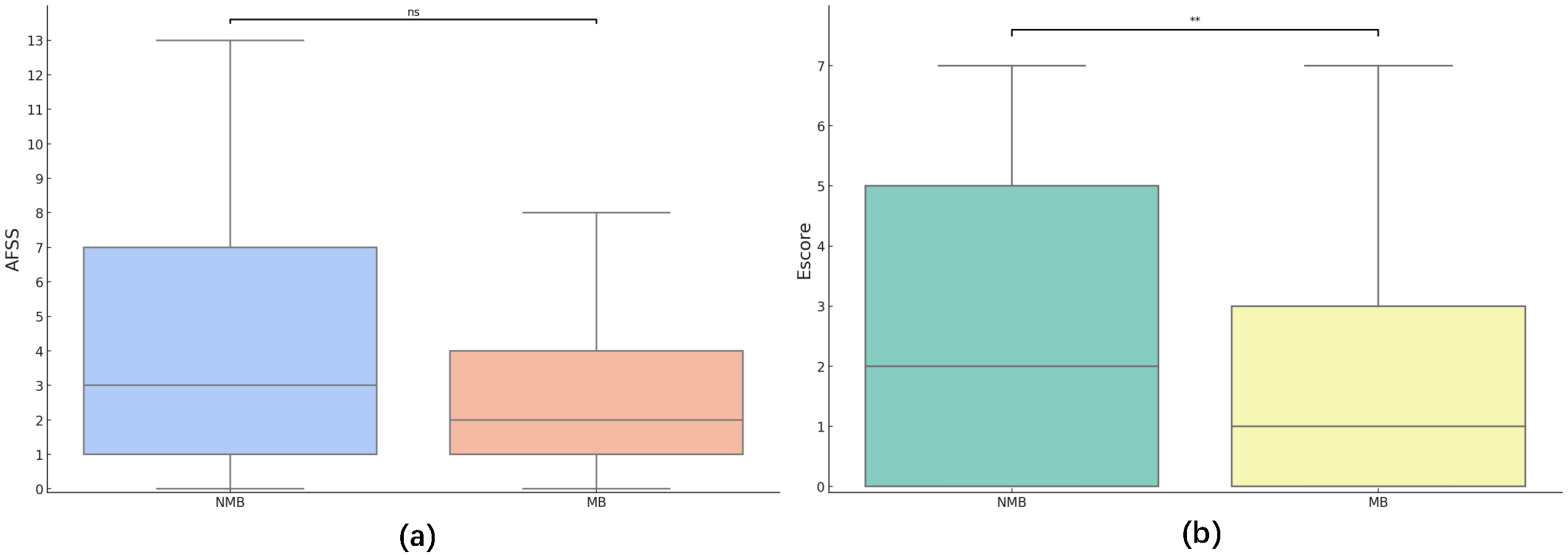
**(a)** Analysis of between-group differences in AFSS categorized author type. No statistically significant difference in AFSS scores was seen between the MB and NMB groups (z = −1.529, *p* = 0.126). **(b)** Analysis of between-group differences in Escore categorized author type. There was a statistically significant difference in Escore scores between the MB and NMB groups (z = −2.299, *p* = 0.022).

In terms of specialized metrics, there was no significant difference in AFSS scores between MB and NMB groups, but the Escore was notably higher in the NMB group (z = −2.299, *p* = 0.02; Figures 3a,b). Further paired t-tests and regression analysis showed that AFSS scores were significantly higher than Escore in two groups (t-statistic = 17.051/ 10.814, *p* < 0.001/ < 0.001; Table 3A). Using AFSS scores as the independent variable and Escore as the dependent variable in a negative binomial regression analysis, it was found that AFSS scores had a significant positive effect on Escore (Table 3B).

**Table 3A:** AFSS and Escore paired *t* test.

Items	Paired (M ± SD)		Mean difference	*p*	Cohen’s d
	AFSS	Escore			
AFSS paired Escore (MB)	2.97 ± 2.28	1.59 ± 1.80	1.38	0.000**	1.024
AFSS paired Escore (NMB)	3.99 ± 3.38	2.44 ± 2.39	1.55	0.000**	1.127

Within the MB and NMB groups, respectively, the variability of the scores of the two rating scales was tested using paired t-tests, and both the AFSS and Escore showed statistically significant differences (*p* < 0.01), with the mean of the AFSS (2.97/3.99) being higher than the mean of the Escore (1.69/2.44). Cohen’s d value represents the effect size magnitude, where larger values indicate greater differences between groups. An effect size exceeding the threshold of 0.8 (considered large) demonstrates both statistically and clinically significant divergence, reflecting a substantial discrepancy between the two comparison groups. *p < 0.05; **p < 0.01.

**Table 3B:** Negative binomial regression analysis.

Items	Coefficient	Std. Error	z value	*p*	OR	OR 95% CI	McFadden’s R^2^
MB							
Intercept	−1.093	0.164	−6.649	0.000**	0.335	0.243–0.462	
AFSS	0.398	0.036	11.04	0.000**	1.489	1.387–1.597	0.135
NMB							
Intercept	−0.771	0.233	−3.303	0.000**	0.463	0.293–0.731	
AFSS	0.303	0.037	8.084	0.000**	1.354	1.258–1.457	0.147

Dependent variable: Escore. AFSS in both the MB and NMB groups showed a significant positive influence relationship on Escore scores (Coefficient = 0.398/ 0.303, *p* < 0.01), as well as a 1.489/1.354-fold increase in the change in Escore with a one-point increase in AFSS. OR(MB) = 1.489, 95% CI = 1.387–1.597, OR(NMB) = 1.354, 95% CI = 1.258–1.457. McFadden’s R^2^: 0.135(MB), 0.147(NMB) suggests that the effect of AFSS is statistically significant, but the overall explanatory power of the model is not at the desired level (>0.2), and a larger sample needs to be included in the future for a more in-depth exploration. *p < 0.05; **p < 0.01.

In the second part, it was observed that most adequacy scientific videos (50/57, 88%) originate from Bilibili and YouTube. Concurrently, all video platforms were inundated with a substantial number of disqualified videos (252/331, 76%). When categorized by author type, MB creators produced nearly twice as many videos as NMB creators (229:102). Yet, the proportion of qualified videos (Escore ≥ 3) was only 1.47:1 (47:32), while non-compliant videos were 2.6 times higher in the MB group (182:70; Figures 4a,b). Among high-popularity videos (top 30% in Heatscore), the MB group again had more videos (78:21) but with ratio of disqualified to qualified videos, approximately 2:1.

**Figure 4:**
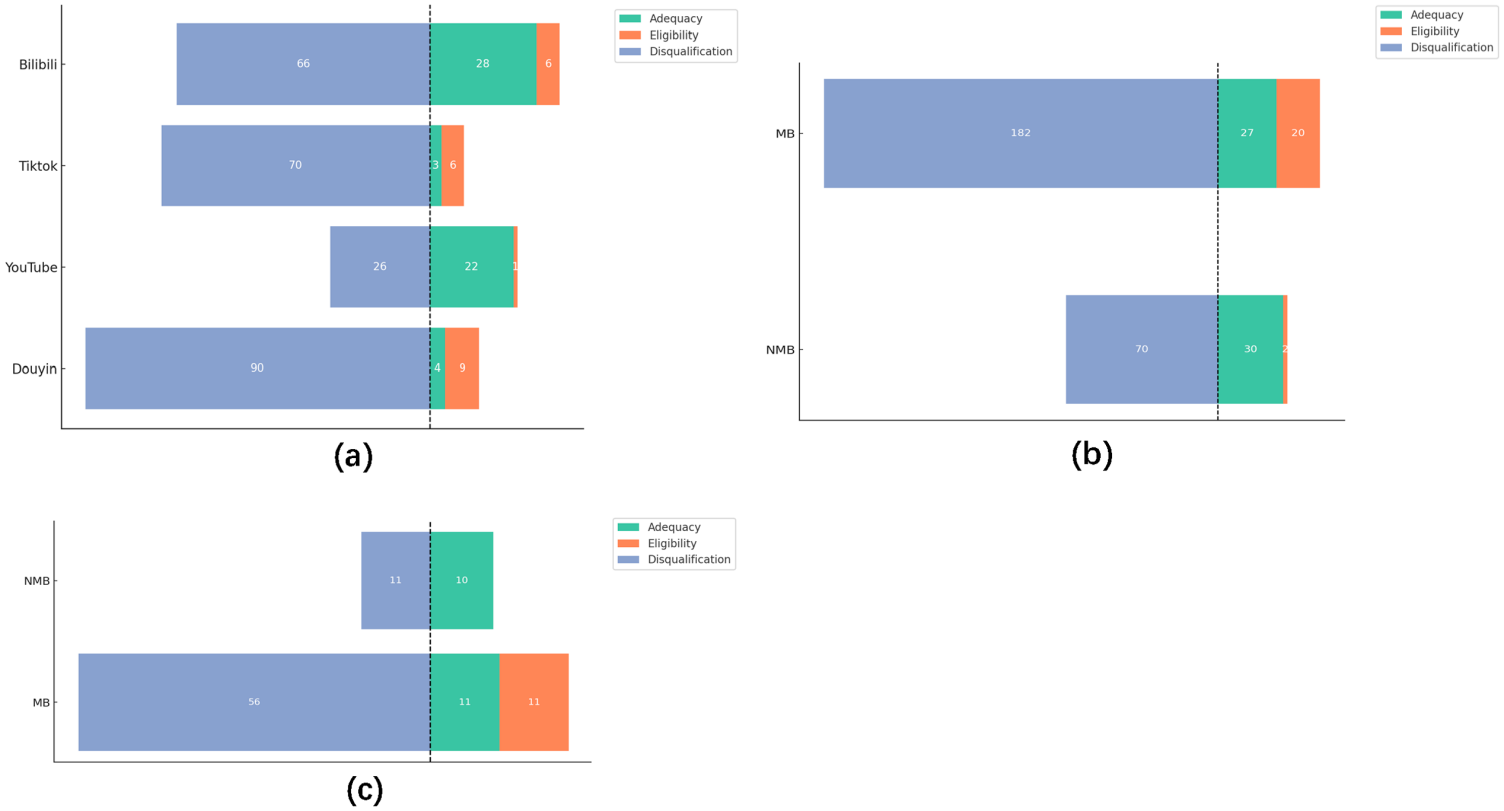
**(a)** Video quality datasets between the platforms. **(b)** Video quality datasets between the author types. **(c)** Video dataset (top 30% in Heatscore) between the author types.

## 4 Discussion

### 4.1 Interaction metrics

In previous similar reports, the data from different platforms were analyzed by directly profiling the number of plays, likes, and replies of the sample video (32–34). However, we recognize that different platforms encompass diverse user bases and content strategies. This variability complicates the interpretation of results, given the disparity in data across platforms (Table 1A). Lookingbil et al. took a randomized permutation test approach to statistically analyze user engagement (i.e., the number of views, likes, comments, and shares) of videos on a single video platform and revealed a significant correlation between the number of publishers’ followers, video likes, and the level of user engagement through linear regression, an approach that should be viewed as an attempt at integrative analysis (23). We believe that direct analysis of data from different platforms can well explain the differences in audience population and policy support of different platforms, but a comprehensive indicator may be needed to make a judgment on the popularity of the video itself. Therefore, our study introduces methods like PCA and normalization techniques for further processing interactive metrics (Table 1B). Importantly, the outcomes of these two statistical approaches yielded notably divergent results (Table 1A; Figure 2a).

The above analysis showed that although YouTube and TikTok outperform Douyin and Bilibili on paper, there is little difference in the Heatscore (popularity) among YouTube, TikTok and Douyin platforms. Bilibili consistently trails behind these platforms in terms of engagement. These findings align closely with similar studies in literature, where videos posted by individuals with medical backgrounds (MB groups) tend to garner higher popularity among viewers (35–37) (Figure 2b).

However, the findings are not cause for celebration. PCA analysis revealed that among the variables examined, the number of comments and replies accounted for 58 and 2% of the total variance in the raw data, respectively. Notably, the median number of replies across all platforms was zero, indicating a widespread lack of publisher responsiveness to viewer inquiries. Moreover, despite TikTok’s highest average view count of 41,200, this figure pales in comparison to the nearly 59 million individuals worldwide affected by atrial fibrillation (AF) (1). This disparity underscores the challenge in effectively reaching and engaging with the expansive AF community through current social media platforms.

### 4.2 Specialized metrics

Several previous studies have evaluated content quality using established scales such as DISCERN, JAMA, PEMAT-A/V, and GQS (32, 33, 38, 39) alongside self-constructed scales based on disease-specific guidelines (35, 40–43). The former has the advantages that the scale has been extensively validated, and the reliability of the conclusions is greater, but the applicability is poorer, and the content of the evaluation lacks the specialization of the disease in which it is related, as opposed to the latter, which is the same in reverse. The situation was such that video platforms were overwhelmed with low-quality and misleading content (41, 44, 45). Hence, specialized scales might be more advantageous in the evaluation of the quality of video content for related diseases, and their disadvantages could be ameliorated through multiple validations. In this study, the AFSS scale was created to cover diagnosis, treatment, and management in accordance with the European and US guidelines for the diagnosis and management of AF. Subsequently, we introduced the Escore scale, designed to prioritize essential and fundamental aspects, reflecting a more necessity approach. In this section, this study had two purposes, firstly to investigate the potential use of a “more basic necessity” rating scale. Secondly, to evaluate whether the content quality of the sample videos was satisfactory.

In developing the AFSS scale, it was found that the detailed scoring criteria, while professional enough, could overwhelm viewers seeking concise information from scientific videos. Hence, the Escore scale was crafted to distill essential elements more comprehensibly.

To assess the Escore scale’s efficacy, we conducted paired t-tests on groups with MB and NMB. Results showed that Escore were significantly lower than the AFSS, it was able to obtain fewer scores than the AFSS, suggesting that the Escore was not a parallel scale to the AFSS (Table 3A). Moreover, negative binomial regression analyses indicated a strong positive correlation between AFSS and Escore scores (Table 3B). The above results indicated that the “basic necessity” Escore scale maintained a high level of consistency and validity with the “complexity and comprehensiveness” AFSS scale. The similar OR valves suggested that the Escore performance is consistent across the two datasets, with no significant variations, supporting the stability of the Escore. As such, the Escore was qualified and potentially useful as a “more basic necessity” scale.

### 4.3 Satisfaction with content quality

In recent years, it has become a common way for the public to learn about diseases through online video sites or software (46–48). Tan et al. found that videos featuring medical professionals, highlighted by titles or attire, tend to attract more engagement (49). However, the findings of several studies are not positive about the quality of video content from professional authors (50–52).

Both publishers and platforms were involved in ensuring the quality of the content of the videos uploaded on platforms. This study assessed the publishers’ by rating the content quality of the sample videos using the AFSS/ Escore scale. It was noted with surprise that the median AFSS scores were only 2.0 and 3.0 out of a total of 13, and that there was no statistically significant difference in the scores between the two groups (Figure 3a). It indicated that the overall content quality of current streaming videos is low, and that the MB group has a higher Heatscore (popularity), but does not have the favorable conditions to create higher quality videos. On the one hand, the MB group may be more likely to be believed by the general public due to their platform accreditation or with the title of medical practitioners, but the quality of their existing videos is not sufficient to provide adequate information to the general public. On the other hand, regardless of whether or not the publishers have a medical background, the overall quality of the video content is substandard, and the incomplete introduction of the treatment content related to atrial fibrillation may result in patients having a biased understanding of the treatment, regardless of the medical background of the publisher. This may lead to compliance problems in long-term treatment. These shortcomings were even more prominent in the Escore scores, where it appeared that publishers in the NMB group, who did not have a medical background, were better able to create “more basic and necessary” scientific videos (z = −2.299, *p* = 0.005; Figure 3b). It is possible that NMB creators may encounter challenges in comprehending the intricate ECG manifestations, electrophysiological changes and disease management of AF. Consequently, these creators may tend to the production of video content that focuses on more readily comprehensible aspects of the disease, such as its manifestations and therapeutic treatments which are more aligned with the Escore scoring. Conversely, for creators in the MB group, the traditional medical education they received—which may dedicate several hours to comprehensively teaching a single disease—forces them to focus only on partial aspects of a condition when producing videos. This limitation stems from three critical constraints: their ingrained educational paradigms, video duration limits, and personal energy reserves. These factors collectively contribute to their challenges in crafting Escore scale based high-quality videos. However, of greater concern to us was the fact that, like the findings from the interactive metrics, overall, the quality of content in both groups remained low.

Next, we categorized the content quality into Adequacy, Eligibility, and Disqualification according to the Escore score range, aiming to figure out whether the platforms have reviewed the quality of the content. The results are consistent with the concerns expressed in related studies (41, 44, 45). Although platforms have already introduced medical certification measures (53–55). Still, video platforms were overflowing with disqualification videos (252/331, 76%), and there were only 14 more eligible videos in the MB group (1.47-fold) but 2.6-fold more disqualification videos in the NMB group when there were twice as many sample videos as in the NMB group (Figures 4a,b). We analyzed the top 30% of videos in the Heatscore to figure out whether platforms are grading and supporting high-quality videos. It was found that even among the MB group videos with high popularity, there is still a large proportion disqualification video (56/ 78, 72%). The MB groups had 3.7-fold videos than the NMB group but had 5.1-fold disqualification video while having only 2-fold qualified video (Figure 4c). Combined with the previous finding that the Heatscore of videos in the MB group was significantly higher than that of the NMB (Figure 2b), we assume that the platform did not have a well re-assessment process of the content quality while providing support to publishers with medical backgrounds, resulting in a situation where videos in the MB group were currently high in popularity, but a large number of disqualification videos still existed.

In the context of Internet globalization, social media platforms have been shown to be faster, more convenient and possess unique social attributes in comparison to traditional means of publicity. This provides a vast fertile ground for the widespread dissemination of disease-related knowledge, but it also breeds ‘bacteria’. It is incumbent upon platforms to provide creators with guidelines for uploading videos, establish a comprehensive background and content review mechanism to eliminate defective and shoddy works, provide views support for well-produced videos with reliable and detailed content, and dynamically monitor the view data of popular science videos so that searchers can find newer and better-quality videos. For the creators, it is essential to adhere to rigorous standards in medical science video production, ensuring that treatments are not selectively or utilitarian introduced. Secondly, the content should be presented in a manner that is more accessible to the general public, with a reduction in text and the adoption of simpler forms of expression such as images or animations.

## 5 Limitation

We only included videos that ranked in the top 50 search results, and it is possible that this strategy does not fully include search terms that may be used by the public. Second, this study attempted to use the Heatscore and Escore, although they were developed based on previous literature and authoritative guidelines and literature, their reliability needs to be explored in subsequent studies.

## 6 Conclusion

In this study assessing the quality of scientific videos on AF knowledge across different video platforms, with four platforms, it appeared that there was insufficient support for high-quality videos and a lack of a rigorous process for reviewing the quality of the content. Despite having medical backgrounds, creators in the MB group did not consistently produce higher-quality videos. Furthermore, this study introduced a normalization method to analyze data, which revealed significant differences between groups, yielding insights distinct from those obtained through raw data analysis. This methodological innovation presented a new way for future studies with larger sample sizes and across multiple platforms. In terms of content quality evaluation, this study pioneered the validation of a “basic and essential” scoring system, designed to better suit public consumption. This innovative approach offered a fresh perspective for future content reviews on video platforms.

The prevailing tendency among the general public to seek information regarding diseases from online sources has become increasingly pervasive. It is incumbent upon platforms to develop vetting standards, optimize recommendation algorithms and establish dynamic monitoring. Creators should consider forming interdisciplinary teams that integrate physicians (to ensure content authority), media scholars (to refine narrative structure), and visual designers (to achieve cognitive load reduction).

## Data Availability

The original contributions presented in the study are included in the article/Supplementary material, further inquiries can be directed to the corresponding authors.
